# Hand-foot syndrome caused by capecitabine: incidence, risk factors and the role of dermatological evaluation

**DOI:** 10.3332/ecancer.2022.1390

**Published:** 2022-05-16

**Authors:** Marina Vieira Rodrigues de Queiroz, Ana Carolina Tardin Rodrigues de Medeiros, Sarah Pires Toledo, Karina Demoner de Abreu Sarmenghi, Vitor Fiorin de Vasconcellos

**Affiliations:** 1 Department of Dermatology, Santa Casa de Misericórdia de Vitória Hospital, Vitória, ES, 29025-023, Brazil; 2 Department of Oncology, Santa Casa de Misericórdia de Vitória Hospital, Vitória, ES, 29025-023, Brazil; a https://orcid.org/0000-0001-5544-458X; b https://orcid.org/0000-0002-6492-3811; c https://orcid.org/0000-0002-6727-356X; d https://orcid.org/0000-0002-4039-808X; e https://orcid.org/0000-0001-8049-1230

**Keywords:** capecitabine, chemotherapy, hand-foot syndrome

## Abstract

Hand-foot syndrome (HFS), or palmar-plantar erythrodysesthesia, is characterised by erythema, oedema and dysesthesia, which can progress to blistering and ulceration. This condition is described as a common adverse effect of the chemotherapeutic agent capecitabine. The study set out to evaluate real-world incidences; assess severity based on clinical criteria, such as local symptoms, dyschromia, erythema, oedema and ulcerations; and associated factors, such as type of solid tumour, chemotherapy regimen, number of cycles, sex, age and Eastern Cooperative Oncology Group Performance Scale of HFS, related to the use of capecitabine. This is a single-centre prospective cohort study carried out jointly by the departments of clinical oncology and dermatology of a university hospital in the southeast of Brazil. The study showed a 34% incidence of HFS, with most cases classified as mild. There was statistical significance in the correlation of the syndrome with sex and performance scores. HFS is the most common and limiting adverse reaction to capecitabine, and causes significant functional and quality impairments in patients with cancer. With this study, we have reinforced the importance of multidisciplinary assessment for early diagnosis and adequate follow-up.

## Introduction

Hand-foot syndrome (HFS), or palmar-plantar erythrodysesthesia, is characterised by erythema and oedema which can progress to blister formation and ulceration [1]. This condition is a common adverse effect of oral fluoropyrimidine, capecitabine, and it is also associated with other chemotherapy drugs [1–3]. Previous studies indicate that about 22%–77% of the patients treated with capecitabine develop HFS to a certain degree [1, 5].

Capecitabine is a prodrug of 5-fluorouracil that is orally administered and commonly used in some solid tumours, such as colorectal, gastric and breast cancers [1, 3, 4]. The pathophysiology of HFS is not well understood [4]. At the cellular level, the toxicity of capecitabine induces keratinocytes’ cell death and the reduction of the corneum stratum present in this condition [4].

Clinically, HFS is characterised by palmoplantar dysesthesia, a clearly demarcated erythema, with or without oedema, desquamation and fissures, and in advanced stages, blisters and ulceration may occur [5]. In individuals with darker skin phenotypes (Fitzpatrick V–VI), the condition may present as macular hyperpigmentation rather than erythema.

A combination of systemic and topical medications can be administered to prevent and treat the syndrome. In severe cases, it might be necessary to reduce the dose of capecitabine or switch to other medications of the same class that are associated with lower rates of the condition [5]. Although it is not lethal, this condition can affect the quality of life and daily activities of patients [2].

This study aims to assess the incidence, toxicity and prevention of HFS triggered by capecitabine in an outpatient clinic in a Brazilian public health tertiary academic institution.

## Methodology

This is a consecutive and prospective cohort study with cancer patients treated at Santa Casa de Misericordia de Vitória Hospital, in Espírito Santo, between October 2020 and May 2021.

Patients aged over 18 years with anatomopathological confirmed diagnosis of solid organ malignancies and initiated with at least one cycle of cancer treatment with a regimen containing capecitabine, either in monotherapy or in combination with another antineoplastic agent, were included.

In addition, relevant clinical and epidemiological variables of the studied population were collected, such as type of solid tumour, chemotherapy regimen, number of cycles, sex and age. The performance status of patients was assessed using the Eastern Cooperative Oncology Group Performance Scale (ECOG-PS).

Patients with previous peripheral neuropathy or diagnoses of dermatological diseases of palmoplantar involvement, such as palmoplantar psoriasis, leprosy, dehydrosis, chronic palmoplantar contact eczema, connective tissue diseases, secondary syphilis, Raynaud’s disease and small vessel vasculitis, were excluded from the study. Patients who completed treatment before starting this project were also excluded from the study.

The main outcome of this study was to determine the incidence and the degree of involvement of HFS according to the criteria of the World Health Organisation (WHO) and the National Cancer Institute (NCI)/Common Terminology Criteria for Adverse Events (CTCAE) 5.0. Also, we conducted subgroup analyses according to clinical and epidemiological features in order to identify potential associated risk or protective features.

Data were analysed statistically, with categorical variables being organised by frequencies and percentages. Associations between qualitative variables and HFS were made using the chi-square test or Fisher’s exact test (in the case of expected values less than 5). When the association was significant (*p* < 0.05), a residual analysis was carried out to verify the categories that contributed to the association. Data were received in an Excel spreadsheet and analysed in the IBM Statistical Package for the Social Sciences Statistics version 27 program.

This study was approved by the ethics and research committee (Process no. 4.308860).

## Results

Out of the 52 participants who had capecitabine prescribed for their cancers, 8 were excluded (6 were lost to follow-up, while 2 refused treatment).

A total of 44 patients were included in the analysis. About two-thirds of patients were female (*n* = 28; 63%) and the median age was 58 years.

The baseline characteristics are summarised in Table 1. The two most prevalent primary sites of neoplastic conditions were colorectal (*n* = 22, 50%; of this, 11 patients had colon neoplasm, 10 rectal neoplasm and 1 sigmoid neoplasm) and breast (*n* = 16, 36.3%). Other locations were 4.5% pancreas, 4.5% gall bladder, 2.2% gastric and 2.2% cholangiocarcinoma.

The incidence of HFS was 34% (15 patients). The commonest neoplasm associated with HFS was malignant breast cancer (46.7%). Others were colon neoplasm (20%), rectal neoplasm (20%), sigmoid neoplasm (6.7%) and pancreas head adenocarcinoma (6.7%). There were no cases of the syndrome in patients with gall bladder neoplasms, gastric adenocarcinoma and cholangiocarcinoma.

Variables significantly associated with HFS included female gender and performance status (ECOG).

As for the clinical presentations, 73.3% developed mild conditions (Grade I) only with dysesthesia, erythema and minimal skin changes, and patients with higher phototypes (Fitzpatrick V–VI) had only hyperchromia. 20% of the conditions were moderate (Grade II) and the study showed only one severe condition (6.7%), as shown in Table 2.

There was no statistical relevance of the occurrence of HFS with the number of chemotherapy cycles, site of primary neoplasm and presence of metastasis. The association of capecitabine with oxaliplatin apparently did not influence the development of the syndrome.

## Discussion

HFS is the most common adverse reaction of capecitabine [6]. Although HFS is not fatal, it can cause significant discomfort and compromised function, especially in elderly patients, and can seriously affect the quality of life [5].

The incidence of the syndrome in this study was 34% and the functional impairment of cancer patients evaluated by the ECOG score showed a positive association with the occurrence of HFS. This suggests that the greater the involvement of the syndrome, the greater the impact on the quality of life. A study by Heo *et al* [8] found HFS in 116 of 179 patients (64.8%) and in 384 of 881 (43.6%) chemotherapy cycles.

Given these numbers, the importance of close follow-up of these patients by their physician is important, preferably, together with the dermatologist, aiming at early diagnosis and treatment. Such follow-up will in many cases prevent dose reductions or discontinuation of treatment [5]. It is important to remember that patient education plays an important role in the management of HFS. Motivation, combined with a good communication system between physician and patient, has consistently proven to be essential in the management of this syndrome [7, 8].

With regard to risk factors, accumulated data suggest that there are regional differences in the tolerability of fluoropyrimidines, with many reports indicating that East Asian patients have a lower incidence of serious toxic effects when treated with fluorouracil or capecitabine-based regimens compared with white patients. Dietary folate intake and pharmacogenomics have been highlighted as possible contributors to these disparities [2]. Several studies have reported that haemoglobin, serum and red blood cell folate levels were important factors for developing the syndrome. Therefore, the incidence of capecitabine-induced HFS may be improved by targeting these clinical predictors [2, 9]. These variables were not evaluated in the present study.

All patients underwent preventive measures such as urea-based 10%–20% moisturising cream, avoidance of contact with irritating agents and on the use of appropriate footwear [7]. The study presented only one severe case (Grade III NCI and WHO), which can demonstrate the importance of preventive measures adopted and joint monitoring with dermatology, allowing for early diagnosis and intervention.

The number of cycles was not directly statistically associated with the occurrence of HFS, which may be a study bias, as most patients in the study received few cycles of capecitabine. For adequate assessment of the relationship between the occurrence of HFS and the number of doses, further prospective studies with patients using the same final number of chemotherapy are needed.

## Conclusion

HFS represents an important impairment of the function and quality of life of cancer patients. This study demonstrates a significant incidence of the syndrome, with the majority consisting of mild to moderate cases. Among the variables evaluated, there is a significant association with female gender and performance scale (ECOG). We reinforce the importance of multidisciplinary assessment for early diagnosis and adequate follow-up.

## Conflicts of interest

The authors declare that there are no conflicts of interest.

## Funding

No funding was provided for this study.

## Institutional review

This study was carried out with the approval of the ethics committee of Escola Superior de Ciências da Santa Casa de Misericórdia de Vitória – EMESCAM (No. 4.308.860).

All authors have read and agreed to its content.

## Figures and Tables

**Table 1.** Association of gender, neoplasia, metastasis, chemotherapy regimen, number of cycles and ECOG score with HFS.

			HFS		Total	*p*
			No	Yes		
Sex	F	*N*	15	13	28	0.022^a^
		%	51.70%	86.70%	63.60%	
	M	*N*	14	2	16	
		%	48.30%	13.30%	36.40%	
Neoplasm	Pancreas head adenocarcinoma	*N*	1	1	2	0.636^a^
		%	3.40%	6.70%	4.50%	
	Gastric adenocarcinoma	*N*	1	0	1	
		%	3.40%	0.00%	2.30%	
	Gall bladder adenocarcinoma	*N*	2	0	2	
		%	6.90%	0.00%	4.50%	
	Cholangiocarcinoma	*N*	1	0	1	
		%	3.40%	0.00%	2.30%	
	Colon neoplasm	*N*	8	3	11	
		%	27.60%	20.00%	25.00%	
	Breast neoplasm	*N*	9	7	16	
		%	31.00%	46.70%	36.40%	
	Rectum neoplasm	*N*	7	3	10	
		%	24.10%	20.00%	22.70%	
	Sigmoid neoplasm	*N*	0	1	1	
		%	0.00%	6.70%	2.30%	
Metastasis	Yes	*N*	11	6	17	0.894^a^
		%	37.90%	40.00%	38.60%	
	No	*N*	18	9	27	
			
			
		%	62.10%	60.00%	61.40%	
Chemotherapy scheme	Capecitabine + oxaliplatin	*N*	9	3	12	0.500^b^
		%	31.00%	20.00%	27.30%	
	Capecitabine	*N*	20	12	32	
		%	69.00%	80.00%	72.70%	
No. of cycles	1–3	N	17	7	24	0.415^a^
		%	58.60%	46.70%	54.50%	
	4–6	*N*	8	7	15	
		%	27.60%	46.70%	34.10%	
	>6	*N*	4	1	5	
		%	13.80%	6.70%	11.40%	
ECOG-PS baseline before treatment	0	*N*	0	4	4	0.000^a^
		%	0.00%	26.70%	9.10%	
	1–2	*N*	28	6	34	
		%	96.60%	40.00%	77.30%	
	3–4	*N*	1	5	6	
		%	3.40%	33.30%	13.60%	

a Chi-square test

b Fisher’s exact test

**Table 2.** Incidence and severity of HFS and its correlation with WHO and NCI CTCAE 5.0 scores.

Severity	WHO	NCI/CTCAE 5.0	Number of patients	%
Mild	Grade I: Dysesthesia/paresthesia, tingling in hands and feet.	Grade I: Minimal skin changes or dermatitis (erythema, oedema and hyperkeratosis), no pain.	11	73.30
Moderate	Grade II: Discomfort when walking and/or holding objects, pain, erythema and oedema.	Grade II: Moderate skin changes (scaling, blisters, bleeding, cracking and erosion) with pain, relative limitation.	3	20
Severe	Grade III: Painful swelling and erythema in hands and feet and around the fingernails and toenails.	Grade III: Severe skin changes (scaling, blisters, bleeding, fissures, erosions and hyperkeratosis) with pain.	1	6.70
	Grade IV: Erosion, ulceration, blisters and severe pain.	_	0	0

## References

[1] Miller KK, Gorcey L, McLellan BN (2014). Chemotherapy-induced hand-foot syndrome and nail changes: a review of clinical presentation, etiology, pathogenesis, and management. J Am Acad Dermatol.

[2] Yap YS, Kwok LL, Syn N (2017). Predictors of hand-foot syndrome and pyridoxine for prevention of capecitabine-induced hand-foot syndrome: a randomized clinical trial. JAMA Oncol.

[3] Fumoleau P, Largillier R, Clippe C (2004). Multicentre, phase II study evaluating capecitabinemonotherapy in patients with anthracycline- and taxane-pretreated metastatic breast cancer. Eur J Cancer.

[4] Chen M, Chen J, Peng X (2017). The contribution of keratinocytes in capecitabine-stimulated hand-foot-syndrome. Environ Toxicol Pharmacol.

[5] Kwakman JJM, Elshot YS, Punt CJA (2020). Management of cytotoxic chemotherapy-induced hand-foot syndrome. Oncol Rev.

[6] Costa JS, Silva GM, Kameo SY (2019). Síndrome mão-pe induzida por quimioterapia: abordagem clínica e epidemiológica de pacientes com câncer. Rev Bras Cancerol.

[7] Marsé H, Van Cutsem E, Grothey A (2004). Management of adverse events and other practical considerations in patients receiving capecitabine (xeloda). Eur J Oncol Nursing.

[8] Heo YS, Chang HM, Kim TW (2004). Hand-foot syndrome in patients treated with capecitabinecontaining combination chemotherapy. J Clin Pharmacol.

[9] Huang XZ, Chen Y, Chen WJ (2018). Clinical evidence of prevention strategies for capecitabine-induced hand-foot syndrome. Int J Cancer.

